# Diffuse Thyroid Calcifications on Ultrasound: A Diagnostic Challenge

**DOI:** 10.1016/j.aed.2025.11.003

**Published:** 2025-11-19

**Authors:** Jane B. Cleland, Elizabeth Zieser-Misenheimer, Joshua Waltonen, Matthew A. Gorris

**Affiliations:** 1 Department of Otolaryngology, Wake Forest University School of Medicine, Winston-Salem, North Carolina; 2 Department of Endocrinology, Wake Forest University School of Medicine, Winston-Salem, North Carolina

## Case Presentation

**Fig. 1:**
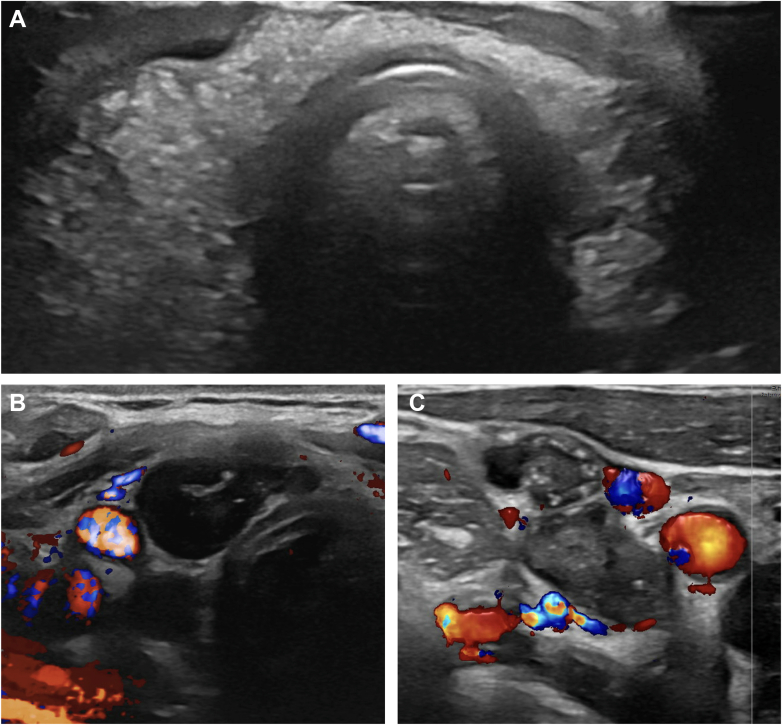
Neck ultrasound showing (*A*) heterogeneous thyroid with punctate echogenic foci. *Right* lobe measures 6.1 × 2.7 × 2.2 cm and *left* lobe measures 5.0 × 1.8 × 1.7 cm. There are also multiple hypoechoic, round lymph nodes measuring up to 1.7 cm in the longest dimension in *right* (*B*) central neck (level VI) and (*C*) lateral neck (level III) with punctate echogenic foci and absent central vascularity.

A 37-year-old female presented with an enlarged right posterior cervical lymph node (LN) for several weeks. She had no systemic symptoms or history of thyroid disease but had a family history of thyroid cancer in her mother and grandmother. Examination revealed an enlarged LN without thyromegaly. Ultrasound (US) showed diffuse thyroid parenchymal heterogeneity (Fig. 1 *A*) and mild to moderately enlarged right cervical LNs (Fig. 1 *B* and *C*), interpreted as indeterminate. Thyroid function was normal (Thyroid-stimulating hormone 2.78 UIU/mL, free T4 0.9 ng/dL) with elevated thyroid peroxidase antibody (TPO 736 IU/mL). Findings were interpreted as Hashimoto’s thyroiditis (HT), and she was referred to endocrinology.

**Highlights**

• Diffuse sclerosing variant (DSV) of papillary thyroid carcinoma (PTC) is a rare, aggressive subtype associated with higher rates of nodal and distant metastasis than classic PTC
• Sonographic findings include diffuse hyperechogenicity, scattered microcalcifications, and ill-defined margins, which may mimic benign or inflammatory disease
• DSV often coexists with or resembles Hashimoto’s thyroiditis, creating diagnostic challenges; suspicious cervical lymph nodes can be an early clue
• Cervical lymph node evaluation is critical—suspicious features such as round shape, irregular borders, absent hilum, and microcalcifications may be the only clue to underlying malignancy

**Clinical Relevance**

This case highlights the importance of lymph node assessment with ultrasound in distinguishing a malignant process such as diffuse sclerosing variant from benign Hashimoto’s thyroiditis, enabling timely diagnosis of an aggressive thyroid malignancy.

**Fig. 2:**
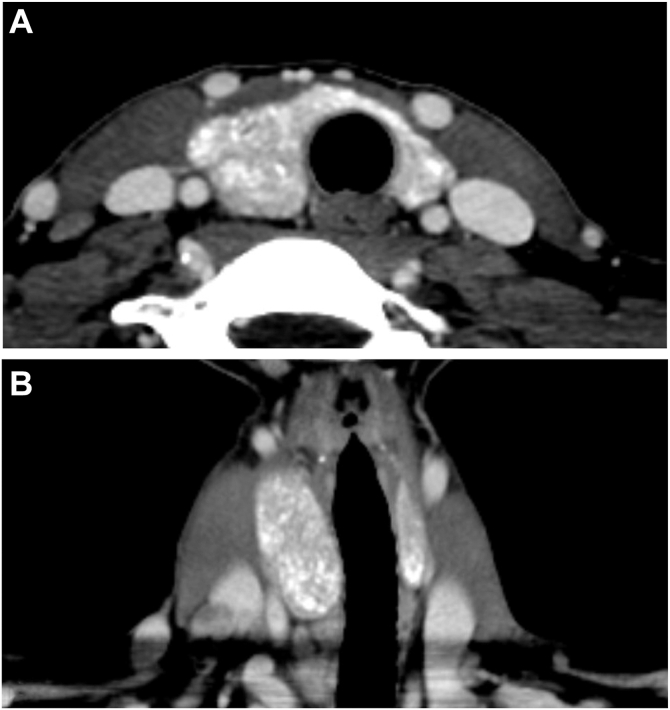
Neck computed tomography (CT) image in the (*A*) axial view and (*B*) coronal view showing asymmetric, heterogeneous mass-like enlargement of the *right* thyroid lobe.

Six weeks later, a repeat US demonstrated a diffusely heterogeneous thyroid and several suspicious LNs. Neck computed tomography revealed diffuse thyroid calcification, asymmetric heterogeneous enlargement of the right lobe (Fig. 2 *A* and *B*), and bilateral cervical lymphadenopathy.

## What is the Diagnosis?

### Answer

Fine needle aspiration of 2 LNs returned suspicious for papillary thyroid carcinoma (PTC). The patient underwent total thyroidectomy with central and bilateral lateral neck dissections, notable for grossly bulky lymphadenopathy in bilateral central compartments and right neck levels 4 and 5. Pathology confirmed diffuse sclerosing variant (DSV) of PTC, characterized by diffuse infiltration of the thyroid (in this case replacing the entire parenchyma) with psammoma body-type calcifications and lymphocytic inflammation. There was extensive lymphatic invasion with metastatic disease involving 19 of 71 LNs across bilateral central and lateral neck compartments. Angioinvasion, extrathyroidal extension, and extranodal extension were not identified. Final staging was pT3apN1b. She recovered without complications and underwent I-131 thyroid ablation (151 mCi).

DSV is a rare, aggressive PTC subtype (0.7% to 6.6% of PTC) characterized by diffuse thyroid infiltration, extensive lymphatic invasion, and nodal metastasis at presentation.^1^^,^^2^ It frequently mimics HT on US, with diffuse heterogeneity, lack of discrete nodules, and presence of microcalcifications (though typically fewer in HT).^3^ Up to 90% of DSV coexists with HT, as in this case with elevated thyroid peroxidase antibody, complicating recognition.^3^

This case highlights the importance of cervical LN assessment in thyroid imaging, as suspicious LNs may be the only clue distinguishing malignancy from HT. Radiologically benign LNs are typically oval (long axis/short axis ratio <0.5), homogeneous, hypoechoic, and contain central fatty hila with vasculature.^2^ In contrast, this patient’s LNs had a round shape, irregular borders, absent central hilum and vasculature, and heterogeneous echotexture with microcalcifications. Other malignant LN features include blurred margins, peripheral vasculature, and hyperechoic echotexture.^2^

A recent analysis by Yang et al demonstrated significantly lower 5-year disease-free survival rates among patients with DSV versus classic PTC after propensity score matching, 69.2% and 93.6%, respectively.^4^ Although DSV exhibits a greater risk of persistent or recurrent disease, there is conflicting data on whether patients with DSV have worse overall survival compared to classic PTC.^5^ Therefore, careful cervical LN evaluation and close multidisciplinary collaboration remain essential to ensure timely diagnosis and optimize treatment outcomes.

## Patient Consent

The patient has been informed about the use of nonidentifying medical information in this case report and has consented to it being published.

## IRB

Reviewed and approved by the medical institutional review board of Wake Forest University Health Sciences IRB IRB00126528.

## Disclosure

The authors have no conflicts of interest to disclose.
